# Cogitations and Vaticinations

**Published:** 1857-05

**Authors:** 


*Cogitations and Vaticinations.* By an Old Fogy.—*The Physiology of the Hebrews—the Pathology of the Greeks—the Chemistry of Dr. Draper.*

More than three thousand years ago the great Lawgiver of the Jews announced the fundamental principle of medicine, *namely*, that the blood is the life of the animal system. I can not just now give the exact words, but this is the exact sense of them. They will be found in the book of Numbers or of some other one of the Pentateuch. "*The blood is the life thereof*"—no doubt about it. But how did Moses learn this? were the learned men of Egypt so far advanced in medical science at that early day as to be able to teach such generalizations as this? I doubt it. About a thousand years later, more or less, Hippocrates taught that altered life, or altered vital action, or diseased action, call it as you please, results from morbid humors in the blood. How remarkably the physiology of the Hebrew and the pathology of the Greek agree! First, of the physiology. And is the blood "the life thereof?" I experiment—I take away the blood from an animal—all that I can get away; and sure enough he is dead. I then reduce this amount in a part as much as I can; and the vital action of the part is lowered or annihilated. The functions of the brain are suspended, and there is a fainting as soon as the heart ceases to send to it its accustomed supply of blood. I look at the parts as they exist in health. Have those the most life in which there is most blood? It is so. The bones have less than the muscles—the cartilages and fibrous tissues less than the brain. I now launch out with comparative physiology. Are these torpid hybernating animals of the lower vertebrata, not possessed of blood as well as the higher animals and man? They are, but their blood is not so highly oxygenated. And what of the non-vertebrata and those animals which have no red blood? are they not alive? They are—but they have an inferior quality of blood, and hence have inferior life. Yes, even the lowest cell has its blood; "it is the life thereof," as much as the crimson current that dashes through the brain of the impassioned orator "is the life thereof." Be not afraid of materialism here; mind or soul is something more than matter, as I shall demonstrate one of these days. I am now speaking of life, which is a very different thing. But even vegetables have life. Yes, and they have too, their blood—and their vital actions correspond to its variations and quantity and quality. It is true, then, as far as observation can go, as far as thought can reach or science can define, that the blood of the whole organized creation is the life thereof. Interrogate nature, from the Behemoth down to the insect of a day—from the cedar of Lebanon to the hyssop on the wall—and the unfailing and unanimous answer will be, "*our blood is our life.*" "The blood is the life thereof," is spouted by the whale amid the surgings of the sea; it is roared by the lion amid African deserts and Indian jungles; howled by jackalls in the streets of deserted cities; bleated and lowed by "the cattle of a thousand hills;" trumpeted by the cock, the clarion of the morning; humm'd by the "shar'd-borne beetle" and his myriad train in the drowsy ear of night; croaked in the baritone of the ominous raven; hymned in a higher key by the ascending lark; sung by the nightingale in the umbrageous grove at eventide; hooted by the solemn owl, and hissed by serpents in their dens. It is whispered audibly in the ear of reason, in the deep abysses of organization which only the microscope can reach; it is chanted by the choristers of spring in green valleys, and screamed by eagles on the mountain crags. The primeval forests teach it. It is spoken forth by the rose of Sharon and the lily of the vale in their budding, in their bloom and their decay; and man and brute, vegetable and animal, health and disease, pleasure and pain, life and death—all organized Nature with her myriad voices, loud as the seven thunders of the Apocalypse, cry *amen* to the words of the prophet.

Of course, changes in the blood would constitute disease according to Moses. Hippocrates thus viewed disease. Let us examine. Can there be any disease without some change, quantitative or qualitative, of the blood? No. But which has the priority—the blood or the solid? Evidently the blood, in nearly every case of disease—unless you choose to call a broken bone or a dislocated joint a disease. The congestions, the inflammations, the anemias, are changes in the amount of blood in the parts—the solids are changed afterwards—the hypertrophies, the atrophies, the growths and deposits, follow previous blood changes, as everybody now knows or will know. The essential fevers are changed qualities or empoisoned states of the blood, as everybody now admits. In what a round-about, ignorant, grandiloquent way the solidists used to explain fever. "The morbific agent being a poison generated by heat, vegetables and moisture, (the fact is, there is very little evidence for this poison, and I vaticinate that it will have but few advocates to commence the next century with,) gets entangled in the spittle and is swallowed--(it could not get into the lungs though already dissolved in the air!)--is swallowed, and acts on the sentient extremities of the nerves, which convey the impression to the brain, which reflects the impression to the heart and organs in sympathy with it, arousing their synergic action by a catenation of morbid links which constitute the totality of the chain of synpathies, direct and reverse, homogeneous and heterogeneous, and thus excite the re-active process and its results in accordance with well known physiological and pathological laws, and which re-active process constitutes the phenomena essential to fever. With this simple statement we proceed." No, you shall proceed no further. Why could you not have said: "The poison being in the air enters the blood through the lungs, and produces the phenomena of fever in a manner not as yet well understood," or have offered any explanation that occurred to you in a few plain words. Words in medicine are like the locusts of Egypt--they darkened all the land.

I propose to say a few words regarding the essential fevers. Nothing better can be expected of an old fogy, than that he should adopt the views of Hippocrates, which may be expressed in three words, *poison*, *coction*, *crisis*; or, to indulge a little more freely, there is a peccant humor, a moteries morbi, which enters the system or is generated in it. This humor causes certain actions and re-actions in the system by which its finally expelled through the various emunctories--excepting always those cases which terminate in death. I accept this doctrine with its *vis medicatrix*. God help us, if there were no *vis medicatrix naturæ!* all the homœopathists would starve to death to begin with, and most of the patients of the regular physician would die; for without the curative power of nature, but few diseases could be cured. We might adjust dislocations, reduce hernias, perhaps repercuss a congestion.

There are three things mainly to be considered about these fevers. The stages of depression, re-action and crisis, by sweat, urine, &c.; all the essential fevers have these stages. The first stage is caused by the presence of the materies morbi in its unconcocted or unoxydized state. It is clear, that such a condition of the blood would diminish that degree of oxydation of the tissues of the organs on which their vital activity is dependent. Hence the langour, the depression, the chill—but this state of the blood is a stimulator of respiration. The presence in the lungs of blood requiring oxygen excites the breathing. Why? why does the kidney secrete urea; It is a first principle we can not go beyond. The unoxydized or imperfectly oxydized peccant humor, then, causes the chill and excites respiration, which introduces an increased amount of oxygen, by which oxygen the peccant matter is oxydized, and re-action or fever is brought about. With the elimination of the concocted or oxydized matter the fever ceases. Doubtless, unoxydized material is also thrown off, and this will explain the fetor of the discharges often observed.

But how do we know that there is a materies morbi? Why, when we see the disease ceasing in thousands of instances, when something has been cast off, as large amounts of urine charged with lithates, or copious sweats, loaded with lactates and fetid matters, as immense ejections and dejections of bile. I say, when we have seen this a thousand times, the conclusion is irresistible that the cause of the trouble was something whose presence constituted the disease, and whose elimination was necessary to the cure. Furthermore, we can imitate these fevers by introducing foreign substances into the blood as pus, the matter of small-pox, &c. We can introduce substances which will cause the phenomena of fever, and then detect them in the excretions; and still further, in these essential fevers the blood is obviously changed—it looks differently from healthy blood. Dncore ; our knowledge of the causes of these diseases forces us to the conclusion that these are morbid matters in the blood. Typhus arises where the crowding is great and the ventilation imperfect, amidst filth and impurities. The blood can not get rid of its effete matters under such circumstances. We handle the materies morbi of small-pox, and introduce it into the system. No one doubts the existence of the materies. Talk about sympathy ! Certainly there is a sympathy between every organ and every other organ ; that is to say, they affect each other in consequence of their connection with each other by blood-vessels, nerves, &c. Sympathy is a fact ; it is not an explanation. Explain to me, dear sir, the sympathy between the kidney and the brain by which narcotism follows, *ischuria renalis*—explain this sympathy ; and what did the grave professor answer? He said, "oh, it's by sympathy!" Yes, the sympathy between the organs is caused by sympathy—and so does opium narcotise by its narcotic properties. Explain to me, the sympathy of A and B. The answer will be, "oh, it is a fellow-feeling, I suppose!"

Having gotten rid of sympathy and sound, let us return to our materies and sense. The materies exist, but what is their nature? what are they? How many kinds are there? These questions are not so easily answered, at least in regard to all the fevers. There is a measly materies in measles, &c., but what is that measly material? What does it come from? The materies of our summer and autumnal fevers, our bilious intermittents and remittents, including yellow fever, appears to me to consist in the carbo-hydrates which the lungs fail to eliminate in the form of carbonic acid and water. I will briefly state the evidences of this proposition : In the first place, bile, which is mainly formed of the carbo-hydrates, is eliminated in large quantities in those diseased, and is evidently itself or in its constituents a part of the materies. Secondly, the liver is allowed to be adjunctive to the lungs in ridding the blood of the carbo-hydrates ; the lungs separating them in the form of carbonic acid and water, and the liver in the form of bile. Of course, when the lungs fail in a degree in this eliminative act, the liver is excited to increased action ; and thirdly, the experiments of Vierordt show, that twice as much carbon is eliminated *per diem* from the lungs in cold weather as in hot—twice as much when the thermometer is at 32° as when it is at 86 or 90°. These experiments certainly explain the bilious disease of hot weather ; nothing more is necessary to show that the carbo-hydrates and bile are the materies morbi of these hot weather fevers. There may be something more than this, but it is not necessary to look further for a materies morbi sufficient to explain the phenomena of these diseases. In yellow fever, it may be urged, that there is often in the worst cases no secretion of bile. True enough, the dark dissolved venous condition of the blood in these cases leads rapidly to congestion, black vomit and collapse. The liver has neither the power nor the time to manufacture bile. A thousand facts might be brought to bear favorably on this theory--the fact that these fevers occur only in hot weather; the fact that no external poison or miasm has been discovered; the fact, that those most exposed to overheating are mostly its victims; the fact mentioned by Dr. Dowler, that the inmates of prisons escape it; the fact mentioned by Dr. Fenner, that bakers are very liable to be attacked; the fact that drunkards and gluttons are almost sure to have the disease, their blood being loaded with carbonaceous matters; the fact that northern men coming to the south are so liable to attack, that the fever has been call the "strangers' fever," but he must be a northern stranger (mind that); the fact that northern folks in general live on a richer respiratory diet than those of the south, because the former need more heating than the latter; the fact that good ventilation is found to be the best remedy; the fact that jack-frost puts a stay to the disease; the fact that—but I have mentioned facts enough, and he who will not believe after considering even these, will not believe "though one rose from the dead." I am bound to write an especial essay on yellow fever before long. Now I will not deny the agency of other things altogether; why should I? who can force me to do it? Dampness, misery, foul air, may aggravate. Decaying vegetables, by absorbing oxygen and throwing off carbonic acid, may impoverish the air of its oxygen, and poison it to some degree with carbonic acid, in various localities. There is no positive malaria necessary. I vaticinate that no one will be found in a quarter of a century from this moment to deny that billious fever and yellow fever differ only in degree.

I shall not have so much to say in regard to eruptive fevers. Their materies it is more difficult to get at; that they consist of waste tissue in process of oxydation may be reasonably presumed. Just think of muscle and nerve as they are dissolved in the metamorphosis of nutrition remaining in a half oxydized state--half-way between tissue and urea. Surely they would constitute materies morbi, and it is an universally admitted fact, that many of these fevers, as typhus and typhoid, arise under circumstances unfavorable to oxydation, as amongst the crowded poor. The waste tissues have to be oxydized before they are eliminated. No doubt that typhus, even admitting it to be contagious, is often generated *de novo.*

Small-pox. Well, it too is generated *de novo.* Where did it come from? Evidently it was generated first in the animal system. It did not tumble out of the fatal box of Pandora amongst the fabled curses of the gods. Introduce a small portion of small-pox matter into the system, and in a week or so this system will furnish a pint of the same matter. Where does the increase come from? from the blood, that's clear. What part of the blood? Why, when you come to look at the blood after all is over, it is the same as it was before; but this makes no difference, a portion of the fibrin or albumen may have been converted into variolous matter and then been reproduced. But this can not be for another reason. viz., the system is after one attack, insusceptible of another, which would not be the case if any of the reproduced elements of the blood furnished the materies morbi. That which furnishes the morbid matter is then something which is passing from the system never to return. Certain circumstances, I know not what, sometimes cause the matter to pass into the variolous transformation, and this matter is capable as a sort of ferment—I say, a sort of ferment, or as a sort of catalytic, of converting to its own nature all similar matters, by contagion, in other systems. But if this decaying matter which renders us susceptible of the disease has already been oxygenized and eliminated, then we will not have it though the contagion be present. If we have but little of it in the system, we will have a slight attack only. The decay of what tissue is it that furnishes the material of small-pox and other contagious fevers which affect the system but once in a life-time? This, no man can tell, and I would merely remark, that Dr. Simon has guessed as ingeniously in regard to these things as any one else. He suggests that the decay of certain organs which are found only in intra-uterine and infantile life, but which pass away as age advances, may furnish the materials of these fevers—as for example, the thymus gland, supra-renal capsules, temporary cartilages, &c., &c. Which one of them furnishes the material for any particular contagion, he does not even venture to guess. All these things will be cleared up

"In a brighter and a better day."

The chemists are to be invoked. Such works as those of Simon, Lehman and Dr. Draper are to be studied. Man, like other material beings, is composed of oxygen, carbon, nitrogen, hydrogen and other elements, whose affinities and re-actions are just the same in him as outside of him. His food is a combustible material in the crucible, and is burned in the blood to give animal heat, just as really as charcoal or wood is burned in a stove to give out heat—that portion of it which goes to form his tissues is oxydized or burned in the performance of the functions of these tissues. The "*vital flame*" is hardly a figure of speech; it is a reality. The atoms yield up their ghosts to swell the vital forces of the system to which they belong. How many thousands of them die to enable the athlete to run a foot-race! How many thousands perish in the orator's brain in the impassioned speech of an hour! Young gentlemen of the medical profession, listen to an old fogy, who can expect to do no great deal himself. His days are pretty much passed. Turn your minds to chemistry, to chemical physiology, if you would thread the labyrinths of pathology and therapeutics. There is no other way. Old fogy as I am—born out of due season—I intend to make the best of my remaining days by following the advice I give to you. Yes, I will learn my alphabet, the elements, and study their combinations, the syllables, and words, and sentences, which make up the great book of nature, and stop only when Death says, "Old Fogy, your task is done! you need not mind living any longer."

Dr. Draper's Physiology—or chemical physiology, as I might term it—recently published, is perhaps the greatest work ever issued from the American press. It ranks with the kindred work of Lehman, but is much more readable and plain. Most of what he has said may be found in other works, but his one volume (I like one volumes!) contains the sense and contents of pretty much all of them. It is especially adapted for students and practitioners. The reading and studying of it, is worth the reading and studying all the medical practices of the last century. I mean what I say, Mr. Editor—read the book and you will agree with me.

Yes, chemistry is the road to certainty in medicine. There can be no difference of opinion on this point. We have started *at the begining* now, unless it turns out that oxygen and carbon are compounds, which I hope will not happen.

It is vexatious to see the little sensation created in the world by men of genius and industry toiling in the noble cause of science ; whilst the noisy politician and courtier, with not half the merit, are gazed upon and lauded by a hemisphere. But it is consolatory to believe that the man of science will live longer, if not so fast, in the memory of his species. Hippocrates will be talked about as long as most of his contemporaries. Bœrhaave will be remembered as long as Peter the Great. Draper's fame will prove as undying as that of the most eminent politician of his age. When he is forgotten there will be nobody living that heard or read of Webster or Clay—and why should this not be so ? What study is more noble than that of man—man in health and man in disease ? Is improvising a few arbitrary laws in the senate chamber nobler than seeking out and following the everlasting laws of Nature ? I trow not. What subject requires more severe study and more power of thought than medicine ? None. The Ciceros, the Burks, the Websters, the Aragos, the Herschells, the mighty men of other Departments of thought and action, could not have done more than has been done by the worthy-to-be-called demigods of our noble profession. The difficulties of other sciences shrink into insignificance when compared with that of medicine; most of them have to be learned preparatory to setting out to explore its wide, wide sea. The thought and research that have been devoted to medicine are sufficient to have made all the discoveries in astronomy, navigation, mathematics, geography and geology, a thousand times over. The good days of the profession are coming. Medicine is to be a science in the highest sense of the term. Quacks, like some ancient reptiles, will be known only as fossils ; and, concerned as we are with the highest interests of Nature's master-piece, our profession will take the highest stand of all things which are purely human and sublunary.—*St. Louis Med. & Surg. Journal.*

